# Prevalence and Treatment of Maternal Substance Use Disorder in Child Welfare

**DOI:** 10.1001/jamahealthforum.2025.0054

**Published:** 2025-03-07

**Authors:** Ezra G. Goldstein, Sarah A. Font

**Affiliations:** 1 Georgia Institute of Technology, Atlanta; 2 Pennsylvania State University, University Park

## Abstract

**Importance:**

Parental substance use is a primary driver of child welfare system involvement, and child welfare services primarily seek to address caregiver challenges, including connecting parents to treatment. Although research highlights that formal child welfare services connect children to health care professionals, less is known about how the system affects caregivers’ substance use treatment.

**Objective:**

To examine the prevalence of maternal substance use disorder (SUD) in the Pennsylvania child welfare system and the association between formal child welfare system response and mothers’ take-up of SUD treatment.

**Design, Setting, and Participants:**

This cohort study used linked Medicaid claims and child welfare system records for mothers enrolled in Medicaid and involved in the Pennsylvania child welfare system between 2015 and 2018, followed by 12 months postsystem contact. The study applied a difference-in-differences framework to estimate associations between child welfare system intervention and maternal SUD treatment utilization. Data were analyzed from January to September 2024.

**Exposure:**

Child welfare system intervention was defined in 3 categories: no formal services, in-home services, and foster care services.

**Main Outcomes and Measures:**

Maternal SUD was defined by *International Classification of Diseases, Ninth Revision *or *Tenth Revision* diagnosis codes. Dependent variables were mothers’ monthly and cumulative use of inpatient and outpatient SUD treatment, defined by procedure and service location codes.

**Results:**

Among 46 484 mothers, the prevalence of maternal SUD was estimated at 62% within the child welfare system population, predominantly involving opioid and polysubstance use. Compared with mothers who did not receive a formal child welfare system response, those who received a formal intervention had a statistically significant higher probability of monthly outpatient (in-home services: 24%; 95% CI, 18%-28%; foster care: 63%; 95% CI, 55%-75%) and inpatient (in-home services: 36%; 95% CI, 23%-48%; foster care: 130%; 95% CI, 103%-156%) SUD treatment in the 12 months after referral.

**Conclusions and Relevance:**

This cohort study demonstrates that formal child welfare services can facilitate substance use treatment for caregivers. As states seek to reduce the role of formal child welfare system responses in responding to parental substance use, alternative strategies to engage and retain parents in treatment are needed.

## Key Points

**Question:**

What is the association between child welfare system intervention and mothers’ use of substance use disorder (SUD) treatment?

**Findings:**

This cohort study of 46 484 mothers in Pennsylvania found that a majority of mothers involved with the child welfare system had a SUD but most were not receiving regular treatment leading up to or after system contact. However, a formal child welfare intervention—in-home services or foster care services—was associated with increased probability of outpatient and inpatient treatment for SUD.

**Meaning:**

In the absence of formal child welfare intervention, caregivers may not receive or engage with substance use treatment.

## Teaser

This cohort study examines the prevalence of maternal substance use disorder in the Pennsylvania child welfare system and the association between formal child welfare system response and mothers’ take-up of substance use disorder treatment.

## Introduction

Forty-six million US adults had a substance use disorder (SUD) in 2021,^1^ more than twice as many as a decade earlier,^2^ placing an estimated 9 million children living with a parent with an ongoing SUD^3^ at heightened risk for developmental challenges, neglect, abuse, and even death.^4,5^ Given these elevated risks to child safety, parental substance use is a persistent driver of child welfare system (CWS) involvement,^6,7,8^ and estimates found in the literature from several states suggest that parental substance use is a factor in 27% to 42% of all CWS cases and the majority of foster care placements.^9,10,11,12^

CWS agencies typically address families with substance use concerns through in-home case plans or referrals to voluntary community-based services, reserving foster care placement as a last resort.^13^ Once CWS formally opens a case, whether for in-home services or foster care placement, agencies are obligated to provide services to the family—often including SUD treatment—to reduce future CWS involvement and promote a safe, nurturing environment. This responsibility is stronger in foster care cases, where federal law imposes strict timelines for reunification, prompting agencies to prioritize resources toward their most urgent needs. To fulfill this obligation, CWS agencies connect caregivers to treatment options, secure appointments with treatment specialists, or bypass waiting lists to facilitate access to SUD treatment.^14^

Although recent evidence highlights that formal CWS services can improve health care utilization among children in homes with SUD concerns,^15^ the empirical research on system-involved caregivers with SUD is relatively sparse. Specifically, estimates of the prevalence of SUD among caregivers in contact with CWS vary, with much of the literature relying on retrospective survey data rather than administrative records.^16,17^ Moreover, while recent research indicates that the majority of parents with SUD concerns referred to CWS do not receive SUD treatment,^9,12^ it remains unclear whether treatment utilization varies by CWS response type or changes due to CWS services. States have drastically reduced the rates of foster care entry^18^ and, to a lesser extent, in-home service cases^19^ during a period of growing addiction^20^ and amid evidence of its consequences for children.^21,22^ If formal CWS services impact treatment utilization, then CWS must seek alternative efforts to engage parents with SUD treatment. By combining administrative records from CWS and Medicaid, this study aims to (1) measure the prevalence of maternal SUD diagnoses among CWS-involved mothers and (2) estimate the association between CWS formal responses—whether opening an in-home services case or placing a child into foster care—and SUD treatment utilization.

## Methods

### Data Sources

To estimate the prevalence of SUD among mothers who come into contact with CWS and assess how CWS is associated with changes to SUD treatment, we linked 2 sources of administrative data: individual Medicaid claims from the Office of Medical Assistance Programs for 2008 to 2019 and CWS referrals from Pennsylvania’s Office of Children, Youth and Families between 2014 and 2021. To link individuals between these sources, we used commonly used probabilistic matching techniques. In the eMethods in Supplement 1, we further describe the administrative data sources and record-linking methods.

This study was approved by the institutional review board at Pennsylvania State University. Informed consent was not needed due to analysis of secondary data. The study followed Strengthening the Reporting of Observational Studies in Epidemiology (STROBE) reporting guidelines.

### Study Population

**Figure 1.**
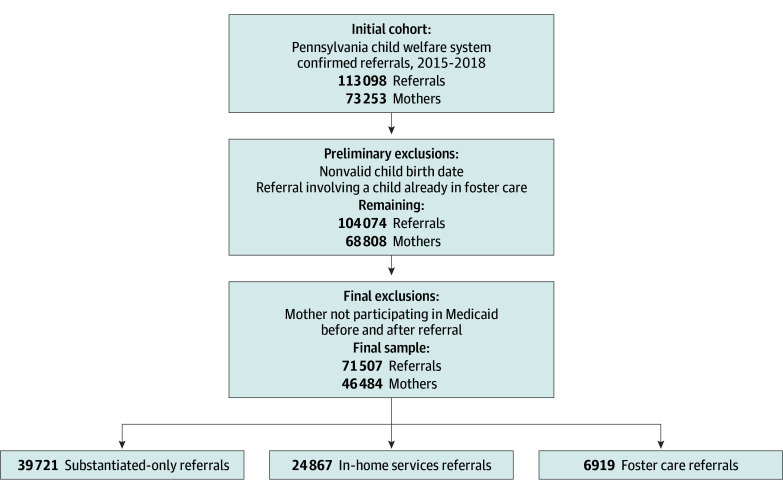
Analysis Sample Selection In the first step of sample selection, 113 098 child welfare system referrals between 2015 and 2018 were associated with 73 253 distinct mothers who could be linked to Medicaid records. In the second step, the child listed in the referral was required to have a valid (nonmissing or implausible) birth date and was not already in foster care. In the third step, the mother listed on the referral was required to be participating in Medicaid both 1 year before and after the referral. Altogether the analysis sample contained 71 507 child welfare system referrals associated with 46 484 mothers. Of these referrals, 39 721 resulted in no formal child welfare system services (substantiation only), 24 867 resulted in formal in-home services, and 6919 resulted in foster care placement.

This analysis focused on CWS referrals between 2015 and 2018, such that SUD treatment outcomes and other instances of CWS contact were observed for a full year before and after an individual CWS referral. Figure 1 displays the analysis sample inclusion criteria, which yielded an analysis sample that included 71 507 CWS referrals pertaining to 46 484 mothers. eTable 1 in Supplement 1 shows differences in demographic and case characteristics for the analytic sample compared with referrals excluded due to the Medicaid restriction; on the whole, included cases disproportionately involved younger children and referrals related to parental substance use.

### Measures

#### CWS Involvement

CWS involvement was measured at each individual referral for an allegation of child maltreatment made to Pennsylvania’s Office of Children, Youth and Families. Referrals include details on the alleged maltreatment, involved parties’ demographics, and case outcomes. Each referral was categorized into 3 levels: substantiation only (allegation substantiated but no case opened for formal services), in-home services (case opened without foster care), and foster care services (case opened with at least 1 child entering foster care). The substantiation-only subgroup differs in that CWS may recommend voluntary services, though participation rates are typically low.^23^ Conversely, cases involving in-home services or foster care feature more active CWS involvement, including caseworker check-ins, tailored service plans, and monitoring SUD treatment when appropriate. Referrals were also classified by alleged maltreatment types, including parental substance misuse, substance-affected infant, physical or sexual abuse, emotional maltreatment, serious physical neglect, other neglect, parent mental health, domestic violence, moral neglect, failure to protect, inadequate supervision, unmet material needs, inappropriate discipline, and serious child-behavioral concerns.

#### SUD Treatment

SUD diagnoses included *International Classification of Diseases, Ninth Revision *or *Tenth Revision* diagnostic codes for substance abuse or dependency, grouped by type: opioids, alcohol, cannabis, cocaine, other stimulants (amphetamines), inhalants, hallucinogens, sedatives, and other or polysubstance use (eTable 2 in Supplement 1). Prevalence was estimated as an ever-to-date measure of SUD diagnoses and was based on diagnoses from 2008 through 1 year post-CWS referral; this estimate is conservative due to the truncated observation period and because mothers may not have been continuously enrolled in Medicaid since 2008.

For caregivers involved with CWS, treatment for SUD typically involves counseling, medication for SUD or behavioral health, or a combination.^24^ We classified SUD treatment into 2 categories: outpatient (including professional office visits) and inpatient. Claims were categorized based on a combination of diagnosis, procedure, health care professional specialty, and place of service codes (eMethods in Supplement 1). For each of the 12 prereferral and postreferral months, we identified monthly treatment (whether mothers received any inpatient or any outpatient treatment in that month) and the prevalence of SUD treatment as the cumulative incidence of treatment (whether the mother had ever received that treatment type between January 2008 and the present month). A supplemental analysis of medication-assisted therapy for opioid use disorder is available in eFigure 1 in Supplement 1, though we also later discuss the limitations of the analysis.

### Statistical Analysis

The analysis sample was structured to observe SUD diagnosis and treatment within the 12 months prior to and after an individual CWS referral (eMethods in Supplement 1) and consisted of 1 818 025 mother-by-referral-months. We calculated the prevalence of SUD diagnoses for mothers 12 months following a referral by CWS response and by whether the referral included an allegation of substance use. We also calculated the raw trends of SUD treatment before and after CWS referral and by CWS response.

To examine the association between CWS response and mothers’ use of SUD treatment, we implemented a difference-in-differences analysis using multivariable linear regression. This model, formally described in the eMethods in Supplement 1, compared treatment utilization in each of the 12 months before and after a CWS referral and between referrals that resulted in in-home or foster care services (2 exposure groups) and substantiation only (unexposed group).

In the main models, key variables included interaction terms between indicators for the month relative to a referral (−12 to 12 months) and indicators for in-home services or foster care response. Models controlled for mother and child demographics and CWS referral characteristics and included fixed effects for the year and county of the referral. Race and ethnicity among mothers and children were collected from administrative data, given evidence that SUD treatment utilization varies by caregiver race,^9^ and included Hispanic, non-Hispanic Black, non-Hispanic White, and other race or ethnicity (including Asian, Native American, and other race or ethnicity, which were combined owing to small sample sizes). Standard errors were clustered by mother, and *P* ≤ .05 was considered statistically significant. Estimates were scaled by the pre-CWS referral mean of the substantiated-only group to interpret estimates as percent differences. We also examined heterogeneity by referral substance use allegations, mother’s race, and child’s age, averaging the CWS response and SUD treatment association across the postreferral period (eMethods in Supplement 1).

The fundamental assumption of the approach is that SUD treatment among mothers in the in-home and foster care services groups would have evolved similarly to those with a substantiated-only referral had their referral received that same disposition.^25,26^ We evaluated this assumption by testing for differences prior to CWS contact (eTable 3 in Supplement 1) and by assessing the robustness of the main findings to alternative specifications and samples in the eMethods in Supplement 1.

Analyses were conducted using Stata, version 17 (StataCorp). Data were analyzed from January to September 2024.

## Results

Of the 71 507 CWS referrals included in the analysis sample, 55% were substantiated only, 35% received in-home services, and 10% resulted in foster care. Of 56 661 children in the analysis sample, 10% were Hispanic, 22% were non-Hispanic Black, 64% were non-Hispanic White, and 4% were another race or ethnicity. The age of the youngest child was younger than 1 year in 20% of referrals and 10 to 17 years in 26% of referrals. Thirty-six percent of referrals involved concerns about parental substance use. For a complete breakdown of the analysis sample by CWS response, see eTable 4 in Supplement 1.

**Figure 2.**
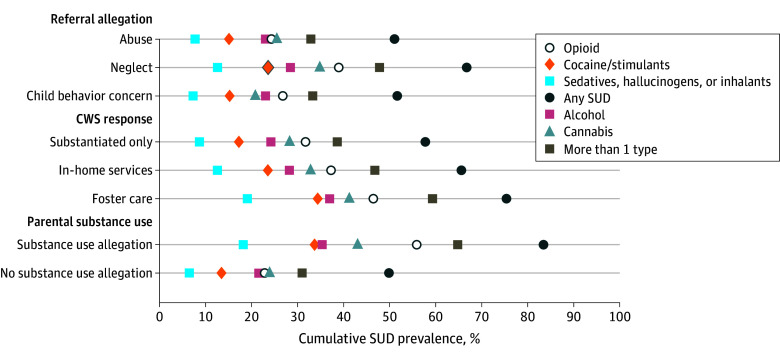
Cumulative Substance Use Disorder (SUD) Prevalence by Child Welfare System (CWS) Involvement The figure presents the prevalence of SUD diagnoses among mothers 1 year following an individual referral. In each set of proportions, the figure shows the share of each group with any SUD diagnosis or a type-specific diagnosis to date.

Figure 2 describes the prevalence of SUD diagnoses among the 46 484 included mothers 12 months following a referral by the allegation category investigated by CWS, CWS response, and by whether the referral included allegations of substance use. The overall prevalence of maternal SUD was estimated at 62% within the child welfare system population. The prevalence of maternal SUD was 52% among cases involving allegations of abuse or those for allegations of child behavior concerns and was 66% for cases involving child neglect. Across response types, maternal SUD prevalence exceeded 57%, reaching 75% in the foster care subgroup. The incidence of multiple SUDs exceeded that of any specific type of SUD, though opioid use disorder was the most common type-specific diagnosis. Lastly, even among mothers who were referred to CWS without any allegations of substance use, the prevalence of SUD reached 50%, with 32% having SUD diagnoses involving multiple substances.

**Figure 3.**
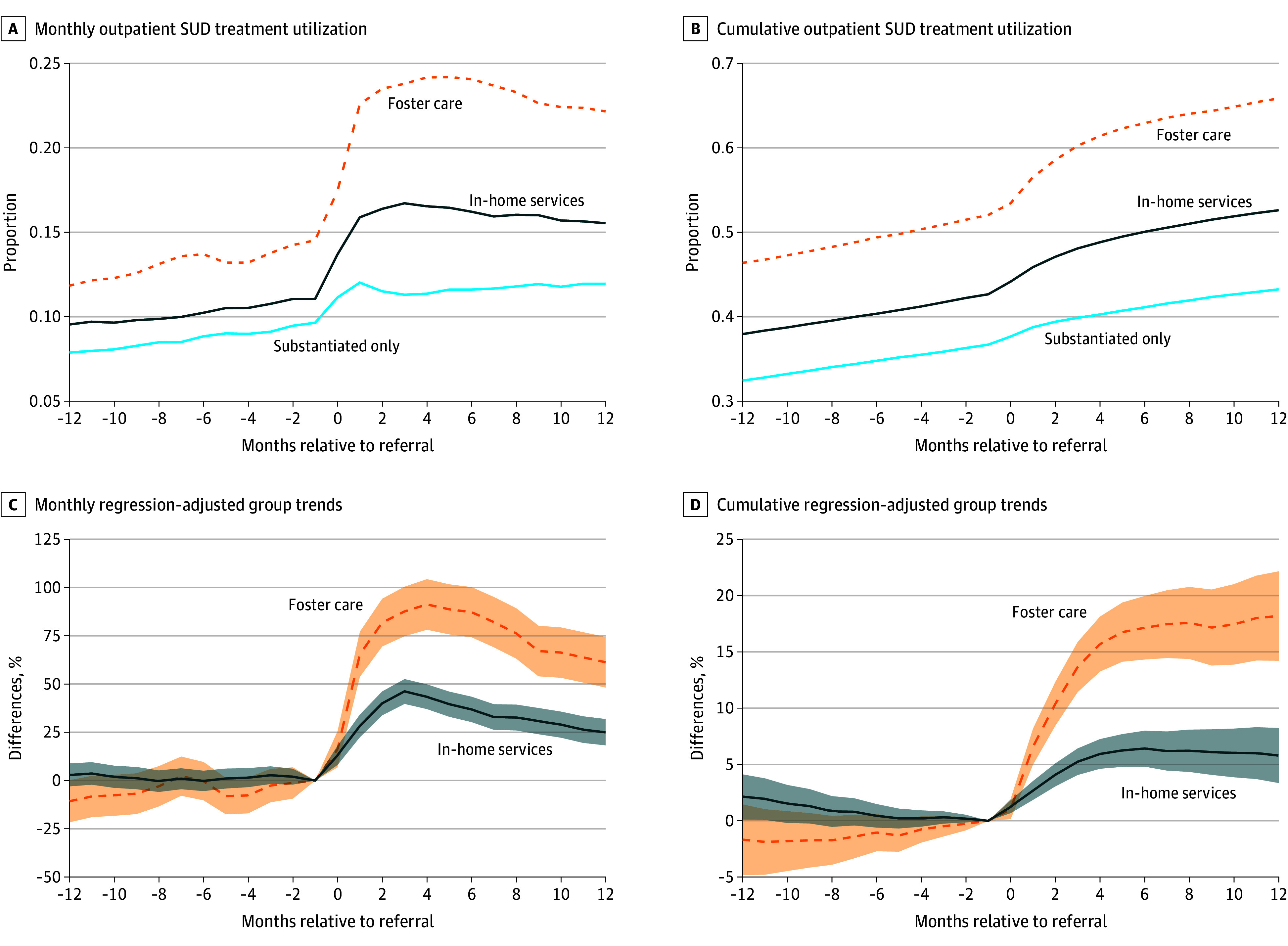
Outpatient Substance Use Disorder (SUD) Treatment by Child Welfare System Response Monthly (A) and ever-to-date (B) outpatient SUD treatment utilization are shown by the child welfare system response group, with estimated differences in treatment between formal services and substantiation only for each month (C) and ever to date (D) leading to and immediately following the referral also calculated (eMethods in Supplement 1). The month prior to the referral is omitted so that estimates are relative to the substantiation-only response group in the month prior to child welfare system contact. Estimates are scaled relative to the mean of the substantiation-only subgroup prior to the month the referral began, such that the y-axis is a (percent) difference relative to the substantiation-only subgroup. The shaded areas represent 95% CIs with standard errors computed by clustering by individual mothers.

To examine how CWS response was associated with SUD treatment, changes in the uptake of both outpatient and inpatient treatment were assessed. Figure 3 plots 4 panels. In each, the x-axis is the month relative to the referral, beginning 12 months before and ending 12 months after. Figure 3A and B show the proportion of monthly and ever-to-date treatment utilization, respectively, by the CWS response group. Three descriptive features emerged. First, there were notable level differences between mothers’ treatment and their CWS response. In each month leading up to the referral, 12% to 14% of mothers received treatment in the foster care subgroup vs approximately 8% and 10% in the substantiated-only and in-home services groups, respectively. Additionally, 52% of the foster care subgroup, 42% of the in-home subgroup, and 36% of the substantiated-only subgroup received any treatment prior to the CWS referral. Second, despite level differences, each group followed similar trends in the period 12 to 1 months before a referral. Finally, each CWS response was associated with an increase in treatment, and the change was largest for the foster care subgroup.

Figure 3C and D formalize these comparisons and plot the resulting differences between substantiation-only cases and formal (in-home or foster care) services across monthly SUD treatment and ever-to-date treatment, respectively. The estimates are scaled so that the y-axis presents the (percent) difference between the in-home services and foster care subgroups relative to the substantiated-only subgroup. Importantly, the figure also presents evidence for the primary assumption of similar trends. For both monthly and ever-to-date SUD treatment, the average estimated differences prior to a referral and relative to the substantiated-only response were near zero and statistically insignificant (eTable 3 in Supplement 1).

A foster care response, compared to substantiation only, was associated with increased maternal SUD treatment utilization by 65% (95% CI, 53%-77%) in the first month following the referral. The foster care subgroup experienced a continuous relative rise in treatment utilization through the fourth postreferral month, when there was a 91% (95% CI, 78%-104%) higher probability of SUD treatment compared with the substantiated-only subgroup. After month 4, treatment gaps stabilized through month 7 and then narrowed. In the 12th month postreferral, the foster care subgroup had a 61% (95% CI, 48%-74%) higher probability of receiving SUD treatment and an 18% (95% CI, 14%-22%) higher probability of having ever received treatment.

An in-home services response was also associated with increased monthly and cumulative treatment utilization, though to a lesser degree. For example, the relative difference between the in-home services group and the substantiated-only group in the probability of monthly SUD treatment was 28% (95% CI, 22%-34%) in month 1, 43% (95% CI, 37%-50%) in month 4, and 25% (95% CI, 18%-32%) in month 12. By month 12, the in-home services subgroup had a 6% (95% CI, 3%-8%) higher probability than the substantiated-only subgroup of ever having received SUD treatment.

**Figure 4.**
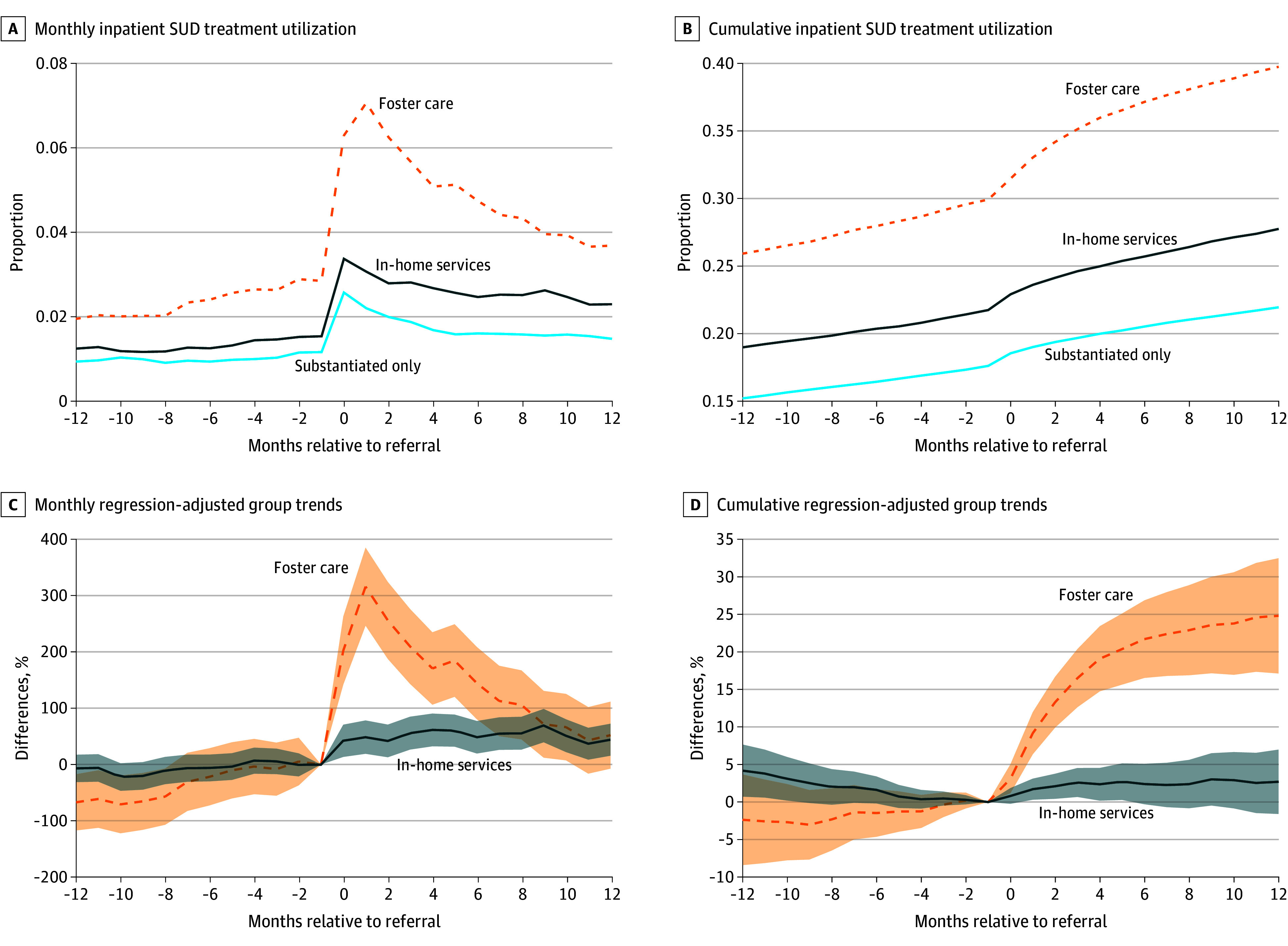
Inpatient Substance Use Disorder (SUD) Treatment by Child Welfare System Response Monthly (A) and ever-to-date (B) inpatient SUD treatment utilization are shown by the child welfare system response group, estimated differences in treatment between formal services and substantiation only for each month (C) and ever to date (D) leading to and immediately following the referral also calculated (eMethods in Supplement 1). The month prior to the referral is omitted so that estimates are relative to the substantiation-only response group in the month prior to child welfare system contact. Estimates are scaled relative to the mean of the substantiation-only subgroup prior to the month the referral began, such that the y-axis is a (percent) difference relative to the substantiation-only subgroup. The shaded areas represent 95% CIs with standard errors computed by clustering by individual mothers.

Figure 4 shows inpatient SUD treatment outcomes and is interpreted the same as Figure 3. Patterns between substantiation-only and formal CWS response groups were consistent, with foster care placement associated with an increase in probability of inpatient treatment of 315% (95% CI, 247%-385%) in the first month postreferral. Since inpatient treatment is typically short term and initiated early in the recovery process, these increases were concentrated in the initial months, tapering off over 10 months and becoming statistically insignificant by the 1-year mark. In contrast, in-home services were associated with an increase in inpatient treatment of 49% (95% CI, 19%-78%) in the first month and remained stable throughout the 12-month period. By month 12, the cumulative probability of inpatient treatment was 25% (95% CI, 17%-32%) higher for the foster care subgroup compared to the substantiation-only subgroup, while the in-home services subgroup showed no statistically significant difference.

Heterogeneity in SUD treatment changes was examined across characteristics that may influence CWS decision-making and treatment responses. First, whether the referral included substance use allegations was analyzed, as these allegations likely shape the focus and type of CWS intervention.^24,27^ Second, differences by race were explored, given evidence that SUD treatment utilization varies by caregiver race.^9^ Finally, whether the child listed on the referral was an infant was examined, as young children are more likely to have substantiated maltreatment allegations and enter foster care.^18,19^

**Figure 5.**
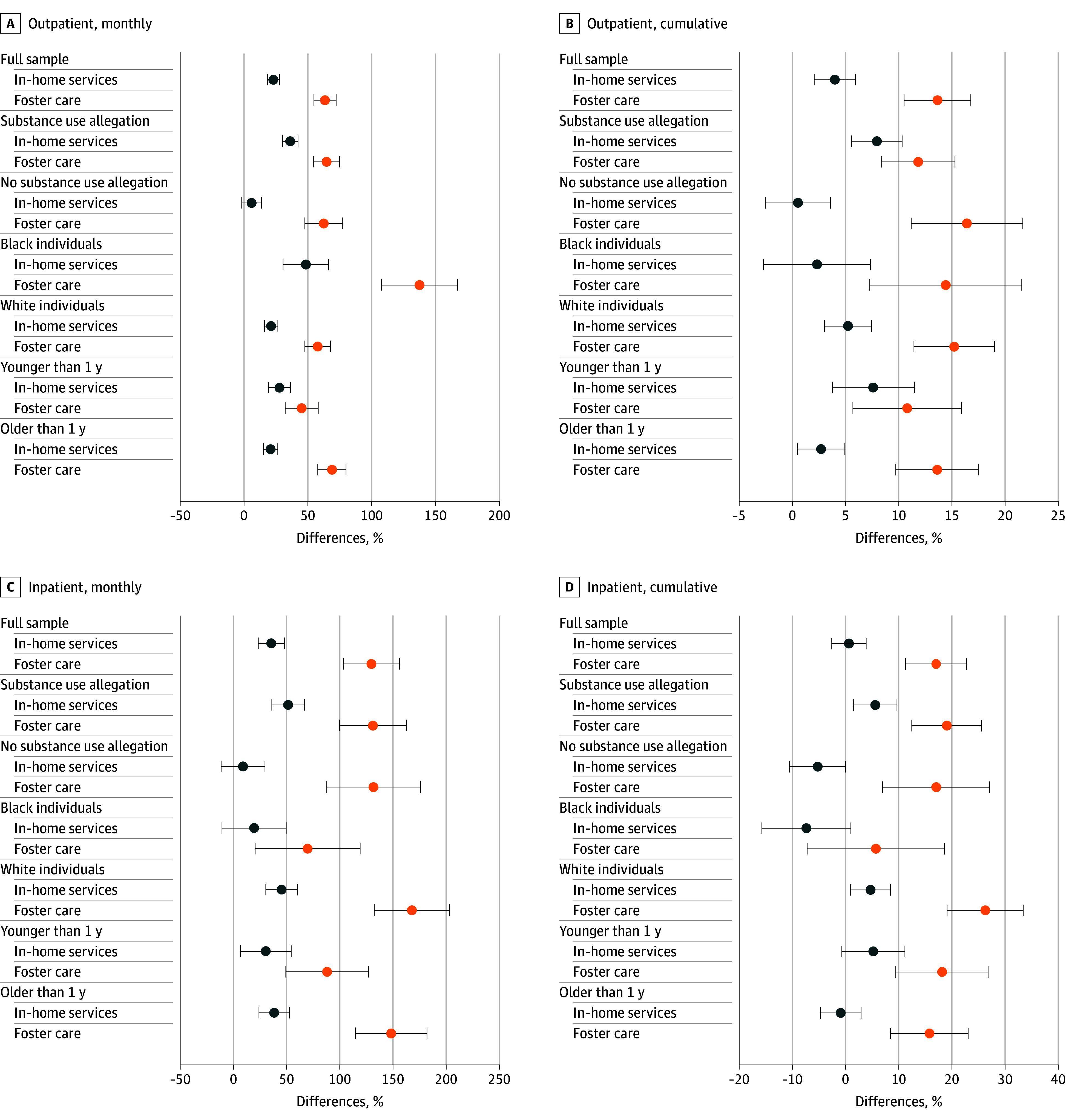
Substance Use Disorder Treatment by Child Welfare System Response, Subgroup Heterogeneity The figure presents the average changes in substance use disorder treatment by formal child welfare system response relative to substantiation only and by subgroup heterogeneity. The estimates are presented across 6 subgroups: whether the child welfare system referral contained an allegation of substance use, separating mothers by whether they were Black or White, and the age of the child on the maltreatment referral (eMethods in Supplement 1). For each subgroup, the estimates after child welfare service referral are averaged and plotted as a single point estimate for each response type (in-home services and foster care), with error bars indicating 95% CIs. The estimates, along with 95% CIs, come from a model that interacts a single dummy variable for the postreferral period with the subgroup of child welfare system response.

Figure 5 presents heterogeneity analyses, where the post-CWS coefficients are averaged and plotted as a single point estimate for each formal response type (see the eMethods in Supplement 1 for specification details). The models showed similar patterns across subgroups, with 3 notable exceptions. First, foster care was associated with both increased outpatient and inpatient treatment (monthly and cumulative) even when the referral did not involve substance use; foster care was associated with raised monthly outpatient treatment of 65% (95% CI, 55%-75%) for referrals alleging substance use and 62% (95% CI, 48%-77%) for those not alleging it. In-home services were associated with increases in monthly outpatient treatment of 36% (95% CI, 30%-42%) when substance use was alleged but was not statistically significant otherwise. Second, the association between CWS intervention and outpatient treatment were larger for Black children (foster care: 137%; 95% CI, 108%-167%; in-home services: 48%; 95% CI, 31%-66%) than White children (foster care: 58%; 95% CI, 48%-68%; in-home services: 21%; 95% CI, 16%-26%), while increases in inpatient treatment estimates were higher for White children (foster care: 167%; 95% CI, 132%-203%) than Black children (foster care: 70%; 95% CI, 20%-119%), though there was not a statistically significant difference in in-home service estimates by race (White children: 45%; 95% CI, 31%-60%; Black children: 19%; 95% CI, −11% to 50%). Lastly, when the referral involved a child younger than 1 year, treatment utilization differences were smaller and not statistically significant.

## Discussion

Substance use is a primary concern among caregivers and children referred to CWS.^6,7,8^ A broad, multidisciplinary literature suggests that more robust partnerships between CWS agencies and community partners, including health care professionals, can improve child safety and well-being when SUD concerns are present.^9,13,15^ Ensuring families’ access to SUD treatment programs and improving treatment adherence has been identified as a critical need for child welfare and family policy.^28^

However, existing research documenting the intersection of CWS and families with SUD concerns is limited in important ways. Much of the literature focuses primarily on child outcomes,^15^ with less attention given to caregivers with SUD concerns, who are most often the focus of CWS interventions and services.^9,29,30^ Additionally, the prevalence of SUD among caregivers in contact with CWS is often measured via self-reports, and few studies have successfully combined Medicaid with CWS records to better understand the service needs of this population.^16,17^ Although recent research using administrative records has highlighted the rates of SUD treatment among caregivers with CWS contact, it has not thoroughly examined how treatment varies based on the type of CWS response.^9,29,30^ Given the variation in CWS practices and services, understanding how treatment utilization changes in response to different CWS actions is needed but underexplored.

This study directly addresses gaps in the literature by focusing on caregivers, particularly those with SUD concerns, who are the primary recipients of CWS interventions. Using administrative records that link CWS data with Medicaid claims, we documented detailed SUD histories and treatment utilization among caregivers around the time of their CWS contact. This approach allowed us to estimate SUD prevalence across various disorders and CWS response types, as well as to analyze the effectiveness of different CWS interventions in connecting caregivers to treatment. These findings reveal that maternal caregivers involved with CWS have considerably higher SUD prevalence—58% in substantiated-only cases, 65% receiving in-home services, and 75% in foster care—compared to the general US female population (8%).^31^ Even when substance use is not mentioned in the referral, SUD prevalence is 50%. Consistent with prior studies, most caregivers do not receive treatment,^9,12^ but formal CWS responses, particularly foster care, are associated with increased treatment utilization for at least a year, highlighting the critical role of CWS in facilitating access to treatment.

Further research is needed to understand why formal CWS responses are associated with increased SUD treatment. This increase likely results from several factors, which are difficult to separate. Formal services may better connect caregivers to treatment specialists, reserve treatment openings, and reduce barriers to treatment like transportation.^14,32^ CWS involvement might also shift caregivers’ health-seeking behavior, encouraging them to engage in and complete treatment.^29,33,34,35^ In cases involving foster care, legal reunification requirements may motivate treatment adherence.^30,34^ Additionally, CWS case plans often mandate physician visits or enrollment in treatment, further driving participation.

Understanding these mechanisms is essential for refining CWS interventions and ensuring that they effectively connect caregivers to the support they need. By identifying the specific factors within CWS processes that drive treatment uptake, future studies can inform the development of targeted strategies to improve outcomes for caregivers and their children. However, consistent with prior research, this study suggests that CWS may not fully engage parents with treatment unless foster care is deemed appropriate.^9,29,30^ Yet, as state and local CWS agencies have shifted away from formal intervention, including monitored services and foster care placement, these estimates suggest a need to engage parents in treatment options. In this respect, stronger partnerships between clinicians and CWS may help prevent further contact with CWS while identifying and treating SUD concerns without deepening families’ involvement.^30,34^

Efforts to increase collaboration between child welfare and substance use agencies have been active in many states for more than a decade and include family drug treatment courts (which mirror drug court diversion programs in the criminal legal system) and sobriety treatment and recovery teams (START; which emphasize joint supervision, team decision-making, and data sharing between child welfare and SUD agencies).^14,36^ However, because these programs broadly operate within the traditional child welfare framework, their value in reducing the need for CWS to facilitate and monitor services is unestablished, and evidence on how to engage caregivers with treatment without formal CWS interventions is limited. Recent evaluations show that, when families received a plan of safe care from the hospital (rather than a CWS referral) following the birth of a substance-exposed newborn, few mothers were connected with SUD treatment.^37,38,39^ Given efforts to reduce formal CWS involvement, strengthening child welfare–substance use agency partnerships, supporting family engagement in SUD treatment, and evaluating policy alternatives are crucial.

### Limitations

These data are limited to Medicaid-enrolled mothers, though Medicaid covers 77% of mothers with opioid use disorder.^40^ Treatment utilization may be underestimated, as programs outside of the health care system or funded through non-Medicaid sources are not captured, though they may be as effective as conventional therapies.^41^ Additionally, pharmacy claims were unavailable, so while we can examine methadone treatment and related appointments (eFigure 1 in Supplement 1), we cannot track medication-assisted therapy prescriptions.

The present data only include maternal caregivers, excluding fathers and relatives, and cover only 1 state; thus, data may not fully capture the relationship between CWS and parental SUD treatment in general. However, Pennsylvania’s CWS response is similar to the US both on rates of child welfare involvement ( 52.8 and 51.2 per 1000 children, respectively)^19,42^ and foster care placement (4.9 and 5.1 per 1000 children, respectively).^43^ Moreover, US rates of illicit drug use among adults are close to those in Pennsylvania (16.3% and 16.6%, respectively).^20^ Given these similarities, we believe that the association between CWS and parental SUD prevalence and treatment identified in this study is likely to be found in other contexts.

Finally, we cannot determine why CWS involvement, particularly foster care, was associated with increases in maternal SUD treatment. Future research is needed to explore whether gaps reflect differences in parent motivation, access to treatment, or court-ordered plans.

## Conclusions

Despite increases in untreated substance use disorders and associated harms to children,^40^ foster care and other child welfare interventions continue to decline. At the same time, demands for more meaningful partnerships between CWS and health care professionals grow. Whether parents received SUD treatment in the absence of formal CWS intervention was largely unknown. This study provides a rigorous examination of the comparative likelihood of mothers receiving SUD treatment under different levels of CWS involvement, finding consistent evidence that formal CWS interventions are associated with increased likelihood of mothers receiving treatment. As agencies aim to reduce formal CWS responses for referrals involving substance use concerns, efforts to identify and implement public health strategies and policies to better address parental SUD treatment are urgently needed.

## Notes

Supplement 1.
eMethods
**eFigure 1.** Medication Assisted Treatment Use By Child Welfare Response **eFigure 2. **Estimates of SUD Treatment by CWS Response, Referrals Without a Separate Recent Referral **eFigure 3.** Estimates of SUD Treatment by CWS Response, Omitting Medical Personnel Reports **eTable 1.** Analytic Sample Exclusion Comparison **eTable 2.** Substance Use Disorder (SUD) diagnosis codes **eTable 3.** Average Pre-Referral Trends **eTable 4.** Sample Composition

Supplement 2.
Data Sharing Statement
