# Supplementary material for: Prevalence and Treatment of Maternal Substance Use Disorder in Child Welfare
Source: JAMA Health Forum. 2025 Mar 7;6(3):e250054. doi: 10.1001/jamahealthforum.2025.0054 (PMC11889472; doi:10.1001/jamahealthforum.2025.0054)
Supplement: Supplement 1. — eMethods eFigure 1. Medication Assisted Treatment Use By Child Welfare Response eFigure 2. Estimates of SUD Treatment by CWS Response, Referrals Without a Separate Recent Referral eFigure 3. Estimates of SUD Treatment by CWS Response, Omitting Medical Personnel Reports eTable 1. Analytic Sample Exclusion Comparison eTable 2. Substance Use Disorder (SUD) diagnosis codes eTable 3. Average Pre-Referral Trends eTable 4. Sample Composition [file jamahealthforum-e250054-s001.pdf]

Supplemental Online Content

Goldstein EG, Font SA. Prevalence and treatment of maternal substance use disorder in child welfare. *JAMA Health Forum*. 2025;6(3):e250054. doi:10.1001/jamahealthforum.2025.0054

eMethods

eFigure 1. Medication Assisted Treatment Use By Child Welfare Response

eFigure 2. Estimates of SUD Treatment by CWS Response, Referrals Without a Separate Recent Referral

eFigure 3. Estimates of SUD Treatment by CWS Response, Omitting Medical Personnel Reports

eTable 1. Analytic Sample Exclusion Comparison

eTable 2. Substance Use Disorder (SUD) diagnosis codes

eTable 3. Average Pre-Referral Trends

eTable 4. Sample Composition

This supplemental material has been provided by the authors to give readers additional information about their work.

© 2025 Goldstein EG et al. *JAMA Health Forum*.

eMethods

Sample Construction

Data Sources and Probabilistic Linking

Our analysis sample was constructed from a linkage of separate state administrative data sources. The primary set of administrative data come from Pennsylvania's Office of Children Youth and Families (OCYF), and contains the universe of substantiated referrals for child maltreatment concerns. The data, which span 2014 and 2021, contain details about each allegation of child maltreatment listed on the individual referral, all parties involved (e.g., the alleged victims, perpetrators, and parents) including demographic information, and the resulting service disposition. The OCYF data also contain the state's Adoption and Foster Care Analysis and Reporting System (AFCARS) file, which reports bi-annual updates of foster care placement episodes in Pennsylvania from 2000 to 2020. The AFCARS file provides information on foster care entry and exit dates and placement types.

Finally, we received records detailing individual medical claims covered by Medicaid between 2008 and 2019 from Pennsylvania's Office of Medical Assistance Programs. The Medicaid records identify claims by their setting, separately by inpatient, outpatient, and professional offices. Individual claims contain details covering the date and place of service, diagnoses (ICD 9 and 10), and procedures (CPT).

Each source of administrative data also contains fields of identifying information, such as names, dates of birth, and personal identification numbers. Since no unique identifier exists to link records across administrative sources, we employ standard techniques to match individuals. Specifically, we employ a probabilistic matching algorithm (a Fellegi-Sunter model) to establish links between the child welfare referral data and each other administrative source.

Specifically, the process creates links between sources by calculating the probability that records across separate administrative sources belong to the same individual. To do so, the model searches for similarities and minimizes discrepancies across available identifying information such as name, gender, date of birth, and

© 2025 Goldstein EG et al. *JAMA Health Forum*.

other administrative identifiers (e.g., social security number or the state's Department of Public Welfare number). We then define an individual as a match if their calculated probability is above a threshold chosen to minimize the rate of false positive links.

Overall, the probabilistic match performs well; for instance, 94 percent of children identified in foster care are matched to the child welfare records. To validate the accuracy of the matching process, we randomly selected and manually examined 100 records, comparing the identifying information from administrative sources. Only two records exhibited discrepancies in more than three matching fields.

Finally, we drop all records associated with individuals who cannot be uniquely matched to the child welfare referral data. We note the potential for bias due to (un)successful linking is likely negligible since the match rate is consistent across demographic characteristics such as race and gender.

Panel Construction and Sample Restrictions

Our empirical design is to examine how SUD treatment changes in the window around a CWS response using a differences-in-differences framework. To do so, we construct our analysis sample such that we can observe claims associated with SUD diagnoses and treatments in the months surrounding each individual referral. More concretely, for each individual referral, we define a window of 12 months prior to and following the month in which the CWS referral began. For each month, we then observe whether or not the mother listed on that referral received SUD treatment.

Our analysis sample is a panel dataset of individual referrals observed for 25 months each. We make few restrictions so that our panel is not strongly influenced by the sample selection criterion. First, we omit the few cases with implausible or missing birthdates listed for the child. Second, we limit the set of referrals to those not involving a child already in foster care. Finally, we require the mother listed on a referral to have been participating in Medicaid both in the periods before and after the referral began.

Mothers can be listed on multiple referrals across the sample period, since they may have contact with CWS on more than one occasion. As a result, one referral may occur within another's observation window. To test

© 2025 Goldstein EG et al. *JAMA Health Forum*.

whether recent referrals influence our estimates of CWS on SUD treatment, in eFigure 2 we further restrict the sample to referrals without a recent (within the past 12 months) separate referral.

© 2025 Goldstein EG et al. *JAMA Health Forum*.

Coding Medicaid Claims

We constructed variables to represent claims with a substance use disorder (SUD) diagnosis code and those signifying treatment for SUD. Medicaid claims data provide relatively limited information about health care service, and our intent is to condition on diagnosis code to flag services (defined by procedure codes) specific to SUD. For instance, procedure codes for "group therapy" or "individual psychotherapy" pertain to a range of conditions not necessarily related to SUD. Thus, we use the combination of diagnosis and procedure codes to confirm that the service was specific to SUD.

At the same time, we did not include all points of health care contact or procedures attached to a SUD diagnosis code as SUD treatment. We excluded categories of procedures that do not constitute a treatment, such as lab testing or emergency care. There are, however, a range of circumstances in which the designation of the service (as treatment or not) depended on the service provider. For example, some procedure codes are generic (e.g., "office visits for continuing patients") and determination of SUD treatment cannot be immediately inferred. For these cases, we relied on published guidance to capture service provider specific treatment, such as Medication Assisted Treatment (e.g., American Society of Addiction Medicine's Billing & Coding resources) and reviewed the provider codes to exclude services unlikely related to SUD treatment.

In eTable 2, we list each ICD-9 and ICD-10 code prefix used to flag SUD claims. We use these SUD diagnoses to identify the following treatment procedures:

• **Outpatient SUD treatment**: This variable was equal to 1 if the claim included a primary diagnosis of SUD and

1. care was NOT provided in an emergency room or inpatient (residential) setting AND
2. the procedure code pertained to a mental health or substance abuse assessment, case management, psychotherapy, psychiatry, or crisis intervention service

• **Inpatient SUD treatment**: This variable was equal to 1 if the claim included a primary diagnosis of SUD and

© 2025 Goldstein EG et al. *JAMA Health Forum*.

1. claim was in the inpatient file (the state of Pennsylvania provided inpatient claims in a separate file, as designated by revenue code)

OR

2. the place of service was listed as an inpatient hospital or residential treatment facility

• **Medication Assisted Therapy (MAT)**: This variable was equal to 1 if

1. The procedure code was any of the following:

   Buprenorphine, Naltrexone, or Methylnaltrexone: 80348, J0570, J0571, J0572, J0573, J0574, J0575, J0592, Q9991, Q9992, J2212, J2315 (CPT)

   Methadone: 80358, 80340, G6053, J1230, H0020, S0109 (CPT) or HZ91ZZZ (ICD procedure code)

   OR

2. Provider specialty type was “Methadone Clinic”

   OR

3. Primary diagnosis was opioid use disorder AND procedure or E/M code was: 99205, 99212, 99213, 99214, 99215, 90971, H0031, 90832, 90834, 90837, 99408, H0033

© 2025 Goldstein EG et al. *JAMA Health Forum*.

Empirical Approach

Differences-in-Differences Event Study Approach

To examine how different levels of CWS contact affect mothers' take up of substance use treatment, we implement a differences-in-differences framework. Our approach compares changes in SUD treatment before and after a CWS referral and between referrals that resulted in foster care placement, in-home services, or substantiation only (no formal services), while accounting for differences along a rich set of covariates. To do so, we estimate the following event study specification:

$$SUDTreatment_{it} = \sum_{j \neq -1} FosterCare_i \, \gamma_j + \sum_{j \neq -1} Services_i \, \delta_j + X'_{it}\beta + \varepsilon_{it}$$

Our unit of interest, i, is an individual mother on a referral, and t represents the month relative to the beginning of the referral and ranges from -12 (1 year before referral) to 12 (1 year after). The dependent variable $SUDTreatment_{it}$ is an indicator variable that equals one if the individual mother received SUD treatment in month t relative to the referral. The variables $FosterCare_i$ and $Services_i$ identify mothers on a referral with a disposition of foster care placement and in-home services, respectively. The specification includes separate indicators for 12 lags and leads relative to the beginning of an individual referral and for each type of formal CWS response. This allows the model to flexibly estimate changes in SUD treatment relative to the month prior to the referral ($t = -1$) and to referrals with a disposition of substantiation only. Finally, $\boldsymbol{X_{it}}$ is a vector of controls including categorical variables for race, gender, and age of the youngest child on the referral; number of children; type of alleged maltreatment; and separate fixed effects for the county of the referral and the year the referral began. Standard errors are clustered by individual mothers to account for arbitrary correlation across referrals that involve the same individual. Cumulative SUD treatment models employ the above specification with two modifications. First, the outcome changes to ever-to-date SUD treatment by month t. Second, the cumulative models account for linear group-specific trends, represented by interactions between the group indicators and the year-month of the observation.

© 2025 Goldstein EG et al. *JAMA Health Forum*.

The coefficients of interest are the γ_js and δ_js, which represent the average difference of SUD treatment in month j between cases with a disposition of foster care or in-home services, respectively, and cases with a disposition of substantiation only. For example, γ_4 represents the difference in SUD treatment between mothers with a case that resulted in foster care placement and those with a substantiation-only disposition four months after the referral began. Estimating each of the γ_js and δ_js allows us to trace out the differences in SUD treatment over time before and after CWS contact began.

The fundamental assumption of our approach is that SUD treatment among mothers who had their children removed or received in-home services would have evolved similarly to those with a case disposition of substantiation only, had their case received that same disposition (Angrist and Pischke, 2009, Athey and Imbens, 2006). That is, SUD treatment should trend similarly, prior to the beginning of a CWS referral, among mothers who had their children removed or received in-home services and those with a case disposition of substantiation only. This common trend assumption implies that the differences in SUD treatment should evolve similarly before an individual referral. If so, then the γ_js and δ_js should be equal to zero for $j < -1$.

We provide evidence for the common trends assumption by directly estimating the γ_js and δ_js prior to CWS contact. Across our main outcomes, there is little evidence to suggest mothers whose cases received formal services were on different trends relative to mothers who did not receive formal services prior to their contact with CWS. The individual estimates of the pre-CWS trends are small and mostly statistically significant. To provide a summary measure of the pre-CWS trend, eTable 3 presents estimates of the average pre-CWS contact differences (and their corresponding 95% confidence intervals) in outcomes between mothers involved in substantiated-only cases and cases that received formal services. In each case, on average, we can reject that the formal services groups exhibited systematically different trends compared to the substantiation-only group.

We also note that our approach only identifies the average treatment effect on the treated (Angrist and Pischke, 2009, Athey and Imbens, 2006). These estimates are informative for the consequences of moving

© 2025 Goldstein EG et al. *JAMA Health Forum*.

mothers from the in-home services and foster care groups into the substantiated-only group, but may be less informative on the consequences of moving mothers from the substantiated-only group into a formal intervention group. However, these estimates are of primary relevance as formal CWS interventions are scaled back (U.S. Department of Health and Human Services, 2017, 2024).

Heterogeneity Estimation

We also explored how the differential changes in SUD treatment by CWS response may differ by characteristics of the referral or demographics of the mother. To do so, rather than creating individual relative-month indicators, we estimate a model that interacts a single dummy variable for the post-referral period with the subgroup of CWS response. The interaction effect provides an average of the difference between the substantiation-only subgroup and the specific CWS response before and after the referral. We then estimate this model for each subgroup specified. Specifically, we estimate:

$$SUDTreatment_{it} = \beta_0 + FosterCare_i\beta_1 + Services_i\beta_2 + PostCWS_t\beta_3 + X'_{it}\beta_4 + (FosterCare_i \times PostCWS_t)\rho + (Services_i \times PostCWS_t)\omega + \varepsilon_{it}$$

The vector of control variables, X_{it} , is the same as above, as are the indicators for foster care and in-home services response. Now, $PostCWS_t$, is an indicator variable that equals one for all post-CWS referral months. The coefficients of interest are then ρ and ω which provide estimates of the average association between SUD treatment and formal services compared to substantiation-only cases across all post-CWS referral months for foster care and in-home services, respectively. Standard errors are similarly computed by clustering by individual mothers listed on the referral, and $p \leq .05$ is deemed statistically significant.

In Figure 5, we present estimates across six specific subgroups which result from estimating the above equation separately for each subgroup. The subgroups are all generated using the information listed on the referral and are whether or not the referral included an allegation for parental substance use, whether the

child was Black or White, or whether the child listed as an alleged victim of maltreatment was less than or older than one year at the time of the referral.

Supplemental Analyses

We further present estimates of changes in SUD treatment after accounting for separate sample restrictions intended to clarify the assumptions of our analytic approach. Since the unit of analysis is the mother-by-referral, the path of SUD treatment could be contaminated by mothers who have had a separate referral prior to the focal referral. As an example, we would be overestimating the SUD treatment engagement changes for cases that resulted in foster care if each referral that led to foster care had a separate referral with a disposition of in-home services within the past year. In eFigure 2, we restrict our analytic sample to study only those referrals without a separate referral in the year prior to the beginning of the focal referral.

Additionally, if a CWS referral begins due to deviations in SUD treatment, or if an individual changes their SUD treatment as a response to an anticipated referral, our primary assumption of similar trends would be violated. Since medical personnel would be most likely to refer a parent to CWS due to a sudden change in SUD treatment, if at all, we restrict the sample and present estimates that omit referrals made by medical personnel in eFigure 3.

We find our conclusions robust to each concern. Regardless of the proposed threat to our approach, the estimates are similar to those of our preferred, full analytic sample. Taken together, we view the estimates as strong evidence that a formal CWS response, particularly foster care placement, increases parental SUD treatment utilization for at least a year following CWS contact.

© 2025 Goldstein EG et al. *JAMA Health Forum*.

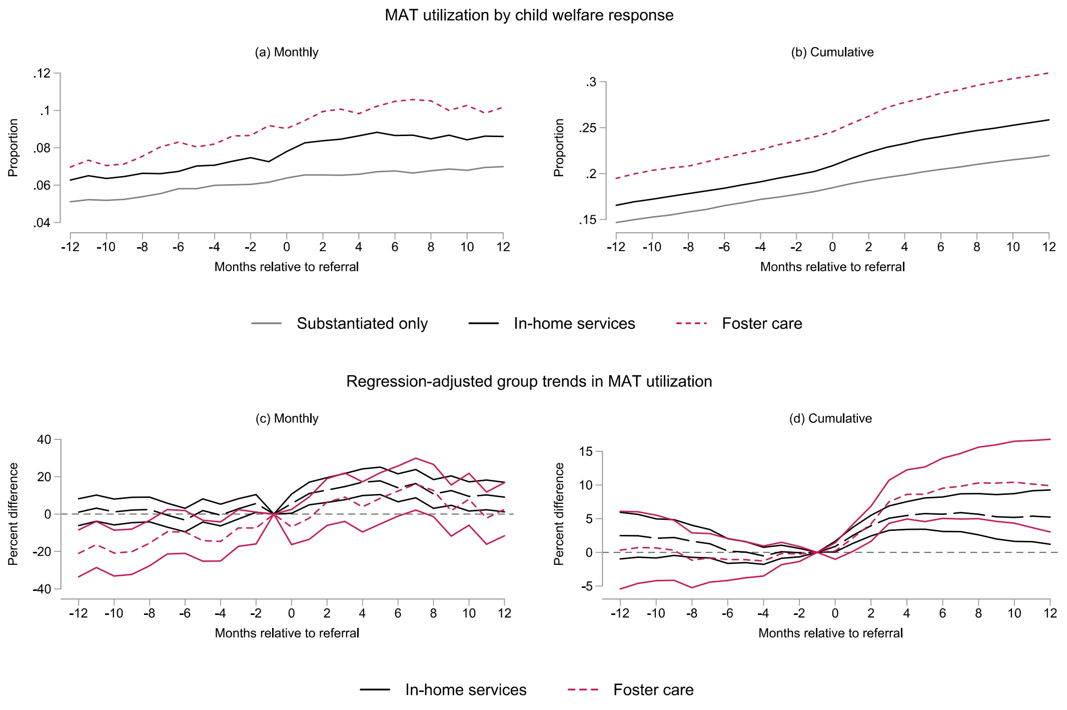

eFigure 1. Medication Assisted Treatment Use By Child Welfare Response

Notes: The top two plots show the proportion of (a) monthly and (b) ever-to-date medication-assisted SUD treatment utilization by the CWS response group. Panels (c) and (d) present estimates of the γ_js and δ_js, for each month leading to and immediately following the referral. The month prior to the referral is omitted so that estimates are relative to the substantiation-only response group in the month prior to CWS contact. Estimates are scaled relative to the mean of the substantiation-only subgroup prior to the month the referral began, such that the y-axis is a (percent) difference relative to the substantiation-only subgroup. The solid lines represent 95% confidence intervals with standard errors computed by clustering by individual mothers. In each panel the x-axis is the month relative to the referral, beginning 12 months before and 12 months after.

© 2025 Goldstein EG et al. *JAMA Health Forum*.

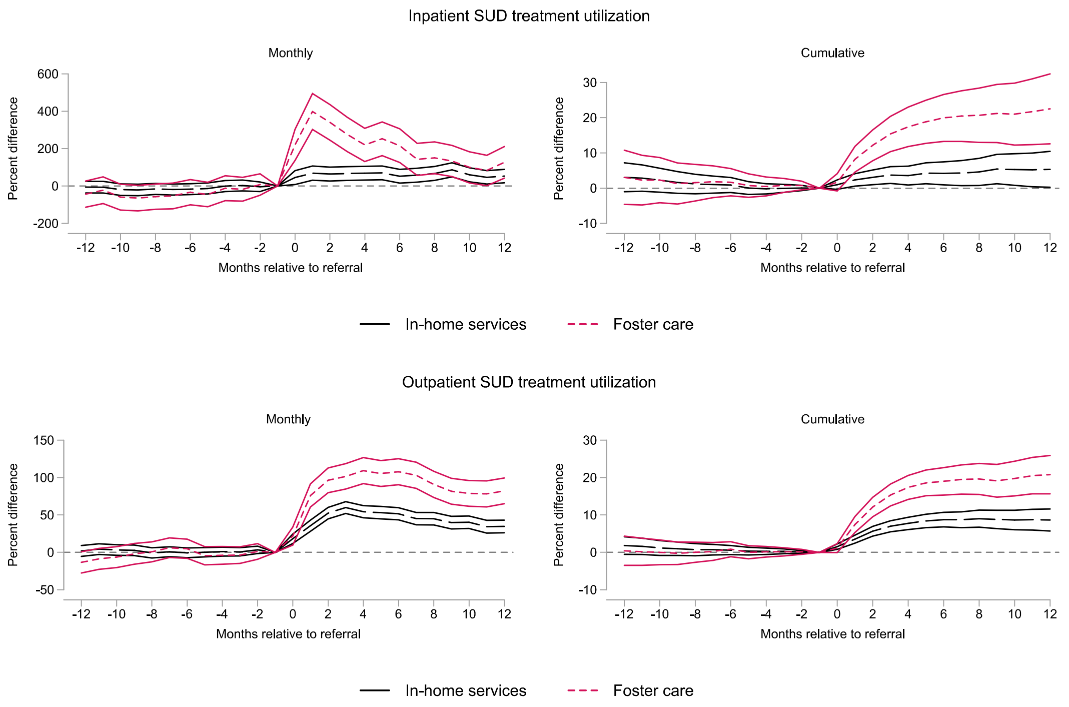

eFigure 2. Estimates of SUD Treatment by CWS Response, Referrals Without a Separate Recent Referral

Notes: The figure plots the estimates of the γ_js and δ_js, for each month leading to and immediately following the referral, and including a sample restriction that limits to focal referrals that did not have another referral within the past 12 months. The top row plots the estimates for monthly and cumulative inpatient SUD treatment, while the bottom row plots the same for outpatient SUD treatment, respectively.

© 2025 Goldstein EG et al. *JAMA Health Forum*.

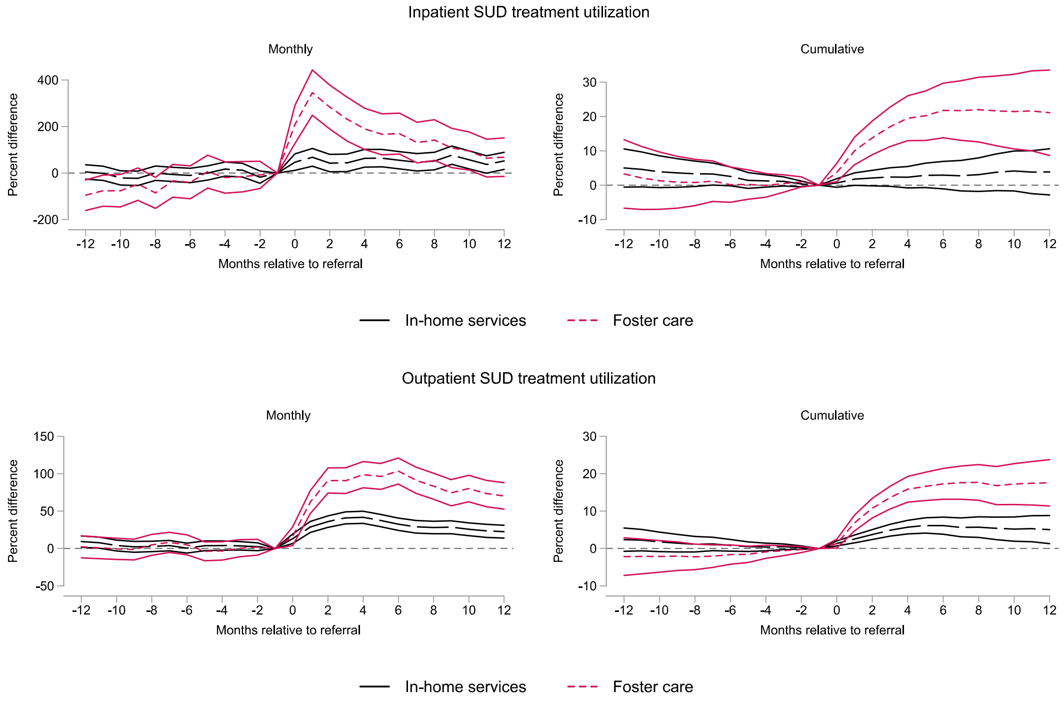

eFigure 3. Estimates of SUD Treatment by CWS Response, Omitting Medical Personnel Reports

Notes: The figure plots the estimates of the γ_js and δ_js, for each month leading to and immediately following the referral, and including a sample restriction that limits to referrals that were not made by medical personnel. The top row plots the estimates for monthly and cumulative inpatient SUD treatment, while the bottom row plots the same for outpatient SUD treatment, respectively.

© 2025 Goldstein EG et al. *JAMA Health Forum*.

eTable 1. Analytic Sample Exclusion Comparison

	Analytic Sample	Excluded (not on Medicaid)
Referral response		
Substantiated Only	55.50	59.89
Accepted for Services	34.84	31.99
Removed w/in 90	9.66	8.13
Race		
White	64.32	66.58
Black	22.45	19.49
Hispanic	10.35	10.53
Other	2.87	3.40
Sex		
Female	48.99	49.48
Male	51.01	50.52
Age youngest child on referral		
<1Y	20.46	13.56
1-3Y	23.39	18.58
4-9Y	29.76	30.58
10-17Y	26.39	37.28
Child Welfare Referral		
Physical abuse	5.45	6.03
Sexual abuse	4.19	6.81
Emotional maltreatment	2.49	2.49
Child-related	19.97	24.59
Serious physical neglect	1.53	1.19
Other neglect	3.72	4.20
Parent substance abuse	36.33	29.48
Parent mental health	11.23	8.33
Domestic violence	8.37	7.83
Moral neglect	0.69	0.84
Failure to protect	16.94	15.30
No available caregiver	2.56	2.77
Inadequate supervision	9.79	8.99
Unmet material needs	23.77	21.68
Inappropriate discipline	7.16	8.22
Substance-affected infant	2.56	2.01

Notes: The table presents the composition of two samples: the analytic sample described in the main paper and the set of referrals excluded due to lack of an indication of Medicaid receipt. Each column presents the distribution of characteristics among referrals included in the corresponding sample.

© 2025 Goldstein EG et al. *JAMA Health Forum*.

eTable 2. Substance Use Disorder (SUD) diagnosis codes

Type of substance	ICD-9	ICD-10
Opioid	304.0, 304.7, 305.5	F11
Alcohol	291, 303, 3050,	F10
Cannabis	304.3, 305.2	F12
Cocaine	304.2, 305.6,	F14
Amphetamines and other psychostimulants	304.4. 305.7	F15
Hallucinogens	304.5, 305.3	F16
Sedatives	304.1, 305.4	F13
Inhalants	N/A	F18
Other (unspecified) and polysubstance use	304.6, 304.7 304.8, 304.9, 305.9, 292	F19

Notes: All diagnoses beginning with the listed 3- or 4-digit short-code/prefix are included.

© 2025 Goldstein EG et al. *JAMA Health Forum*.

eTable 3. Average Pre-Referral Trends

	Outpatient	ETD Outpatient	Inpatient	ETD Inpatient
In-Home Services	0.001	0.003	-0.001	0.003
	[-0.002,0.005]	[-0.001,0.007]	[-0.002,0.001]	[-0.000,0.006]
Foster Care	-0.004	-0.004	-0.004	-0.003
	[-0.011,0.003]	[-0.011,0.002]	[-0.007,0.000]	[-0.008,0.003]

Notes: This table presents the average pre-referral differences (and corresponding 95% confidence intervals) for the four main outcomes and across the in-home services and foster care groups. Each average was calculated by a linear combination of pre-CWS coefficients within each model that estimates the event-study equation.

© 2025 Goldstein EG et al. *JAMA Health Forum*.

eTable 4. Sample Composition

	Full Sample	Subst. Only	In-Home Services	Foster Care
Race/Ethnicity				
White	46775 (64.32)	27486 (68.10)	15384 (60.73)	3905 (55.59)
Black	16328 (22.45)	7571 (18.76)	6485 (25.60)	2272 (32.34)
Hispanic	7529 (10.35)	4068 (10.08)	2747 (10.84)	714 (10.16)
Other	2089 (2.87)	1238 (3.07)	717 (2.83)	134 (1.91)
Sex				
Female	35548 (48.99)	19884 (49.39)	12206 (48.27)	3458 (49.25)
Male	37021 (51.01)	20376 (50.61)	13082 (51.73)	3563 (50.75)
Age at referral				
<1Y	14879 (20.46)	7423 (18.39)	5319 (21.00)	2137 (30.42)
1-3Y	17011 (23.39)	9355 (23.18)	5993 (23.66)	1663 (23.67)
4-9Y	21640 (29.76)	12863 (31.87)	7303 (28.83)	1474 (20.98)
10-17Y	19191 (26.39)	10722 (26.56)	6718 (26.52)	1751 (24.93)
Child Welfare Referral				
Parent substance abuse	26422 (36.33)	14578 (36.12)	9123 (36.01)	2721 (38.73)
Substance affected infant	1865 (2.56)	1081 (2.68)	545 (2.15)	239 (3.40)
Physical abuse	3966 (5.45)	2188 (5.42)	1089 (4.30)	689 (9.81)
Sexual abuse	3047 (4.19)	2369 (5.87)	520 (2.05)	158 (2.25)
Emotional maltreatment	1812 (2.49)	853 (2.11)	774 (3.06)	185 (2.63)
Child-related	14520 (19.97)	7826 (19.39)	5649 (22.30)	1045 (14.88)
Serious physical neglect	1111 (1.53)	498 (1.23)	339 (1.34)	274 (3.90)
Other neglect	2704 (3.72)	1309 (3.24)	1207 (4.76)	188 (2.68)
Parent mental health	8164 (11.23)	4081 (10.11)	3202 (12.64)	881 (12.54)
Domestic violence	6086 (8.37)	3731 (9.24)	1950 (7.70)	405 (5.77)
Moral neglect	500 (0.69)	324 (0.80)	123 (0.49)	53 (0.75)
Failure to protect	12322 (16.94)	6852 (16.98)	4289 (16.93)	1181 (16.81)
No available caregiver	1864 (2.56)	716 (1.77)	643 (2.54)	505 (7.19)
Inadequate supervision	7122 (9.79)	3980 (9.86)	2433 (9.60)	709 (10.09)
Unmet material needs	17288 (23.77)	8840 (21.90)	6819 (26.92)	1629 (23.19)
Inappropriate discipline	5208 (7.16)	3270 (8.10)	1607 (6.34)	331 (4.71)
No prior referral	44165(60.73)	25261(62.58)	15192(59.97)	3712 (52.84)
One prior referral	16820(23.13)	8856 (21.94)	6114 (24.13)	1850 (26.33)
Two prior referals	6757 (9.29)	3559 (8.82)	2382 (9.40)	816 (11.62)
Three prior referrals	2809 (3.86)	1480 (3.67)	962 (3.80)	367 (5.22)
Four or more prior referrals	2170 (2.98)	1207 (2.99)	683 (2.70)	280 (3.99)
SUD Diagnoses in 12 months pre-CWS referral				
No SUD	32891 (45.23)	19786 (49.02)	10706 (42.26)	2399 (34.15)
Multiple-type SUDs	26303 (36.17)	13091 (32.43)	9768 (38.56)	3444 (49.02)
Single type SUD	13527 (18.60)	7486 (18.55)	4859 (19.18)	1182 (16.83)
Opioid SUD	21317 (29.31)	10876 (26.95)	7750 (30.59)	2691 (38.31)
Amphetamine SUD	2793 (3.84)	1406 (3.48)	1017 (4.01)	370 (5.27)
Cocaine SUD	8909 (12.25)	3914 (9.70)	3543 (13.99)	1452 (20.67)
Cannabis SUD	17864 (24.57)	9089 (22.52)	6540 (25.82)	2235 (31.81)
Alcohol SUD	16053 (22.07)	8134 (20.15)	5864 (23.15)	2055 (29.25)
Inhalant sedative or hallucinogen SUD	5703 (7.84)	2485 (6.16)	2281 (9.00)	937 (13.34)
Other/Polysubstance SUD	23494 (32.31)	11535 (28.58)	8852 (34.94)	3107 (44.23)
SUD Diagnoses by 12 months post-CWS referral				
No SUD	27594 (37.95)	17112 (42.40)	8747 (34.53)	1735 (24.70)
Multiple-type SUDs	31471 (43.28)	15507 (38.42)	11806 (46.60)	4158 (59.19)

© 2025 Goldstein EG et al. *JAMA Health Forum*.

Single type SUD	13656 (18.78)	7744 (19.19)	4780 (18.87)	1132 (16.11)
Opioid SUD	25339 (34.84)	12705 (31.48)	9384 (37.04)	3250 (46.26)
Amphetamine SUD	5637 (7.75)	2682 (6.64)	2124 (8.38)	831 (11.83)

Continued on next page

© 2025 Goldstein EG et al. *JAMA Health Forum*.

eTable 4 – *Continued from previous page*

	Full Sample	Subst. Only	In-Home Services	Foster Care
Cocaine SUD	11637 (16.00)	5074 (12.57)	4616 (18.22)	1947 (27.72)
Cannabis SUD	22440 (30.86)	11305 (28.01)	8253 (32.58)	2882 (41.02)
Alcohol SUD	19319 (26.57)	9657 (23.93)	7081 (27.95)	2581 (36.74)
Inhalant sedative or hallucinogen SUD	7794 (10.72)	3367 (8.34)	3107 (12.26)	1320 (18.79)
Other/Polysubstance SUD	26483 (36.42)	12932 (32.04)	10004 (39.49)	3547 (50.49)

Notes: The table presents the sample composition of the analytic sample across several key characteristics. The second column describes the full analytic sample, while columns three through five describe the subsamples of referrals that received a case disposition of substantiation only, in-home services, and foster care placement, respectively. Each column displays the number of referrals and their corresponding share of the sample (in parentheses). Note that some characteristics, such as allegation type, may not be mutually exclusive.

© 2025 Goldstein EG et al. *JAMA Health Forum*.
