# Supplementary material for: Prevalence and Treatment of Maternal Substance Use Disorder in Child Welfare
Source: JAMA Health Forum. 2025 Mar 7;6(3):e250054. doi: 10.1001/jamahealthforum.2025.0054 (PMC11889472; doi:10.1001/jamahealthforum.2025.0054)
Supplement: Supplement 2. — Data Sharing Statement [file jamahealthforum-e250054-s002.pdf]

Data Sharing Statement

Goldstein. Prevalence and Treatment of Maternal Substance Use Disorder in Child Welfare. *JAMA Health Forum*. Published March 07, 2025. doi:10.1001/jamahealthforum.2025.0054

Data
Data available: No

Additional Information
Explanation for why data not available: The administrative data was collected by entering into separate data use agreements with state agencies. As a part of our agreements, we cannot share any of the underlying data. We would be glad to share any code or programs used to generate the results in the manuscript. Moreover, we would be happy to direct any interested researcher to the appropriate state offices necessary to enter into their own data use agreement.
